# Residential Risk Factors for Atopic Dermatitis in 3- to 6-Year Old Children: A Cross-Sectional Study in Shanghai, China

**DOI:** 10.3390/ijerph13060537

**Published:** 2016-05-27

**Authors:** Feng Xu, Shuxian Yan, Qile Zheng, Fei Li, Weihan Chai, Minmin Wu, Haidong Kan, Dan Norback, Jinhua Xu, Zhuohui Zhao

**Affiliations:** 1 Department of Dermatology, Huashan Hospital, Shanghai Medical College, Fudan University, Shanghai 200040, China; xufengdoctor@126.com (F.X.); shuxianyan0225@126.com (S.Y.); feyfey252@hotmail.com (F.L.); 2 Department of Dermatology, The First Affiliated Hospital of Fujian Medical University, Fuzhou 350000, China; ale08172@126.com; 3 Department of Dermatology, Jiading District Traditional Chinese Medicine Hospital, Shanghai 201800, China; chaiweihan@163.com; 4 School of Public Health, Key Lab of Public Health Safety of the Ministry of Education & Key Lab of Health Technology Assessment of the Ministry of Health, Fudan University, Shanghai 200032, China; mmwu@fudan.edu.cn (M.W.); hdkan@shmu.edu.cn (H.K.); 5 Department of Medical Science, Occupational and Environmental Medicine, Uppsala University, Uppsala 75185, Sweden; dan.norback@medsci.uu.se

**Keywords:** dermatology, home environment, redecoration, mold, dampness, urban residency, heredity

## Abstract

*Background*: Atopic dermatitis (AD) is common among pre-school children in Shanghai. This study aimed to identify the risk factors for childhood AD from the perspectives of home environment, demographics and parents-grandparents’ atopic disease. *Methods*: A cross-sectional study was conducted in Shanghai in April–June, 2010. Preschool children’s parents or guardians were invited to participate a questionnaire survey in six districts (two urban and four suburban/rural) and 6624 children were finally recruited (51.3% boys). AD diagnosis was based on the U.K. Working Party’s (UKWP) criteria. Adjusted odds ratios (AOR) with 95% confidence intervals (95% CI) were calculated by multiple logistic regression. *Results*: A total of 8.5% of children ever had AD. Around 10.2% of the mothers had lived in newly renovated/decorated homes (NRDH) during the prenatal period (one year before or during pregnancy) and 9.5% got new home furniture (NHF) during the same period. AD was more common in children when mothers had lived in NRDH homes during the prenatal period (AOR = 1.41; 95% CI 1.03–1.93), the current home had indoor mold (2.00, 1.48–2.70), parents-grandparents’ had atopic diseases (3.85, 3.05–4.87), the children had food allergy (3.40, 2.63–4.40) or children lived in urban area (1.52, 1.18–1.96). Associations between AD and NRDH, NHF and indoor molds were only significant in children without parents-grandparents’ atopic diseases. There was an interaction effect between parents-grandparents’ atopic diseases and NRDH (*p* < 0.05). *Conclusions*: Home renovation/ redecoration, new furniture and indoor mold, urban residency, heredity disposition and food allergy can be risk factors for childhood AD in Shanghai.

## 1. Introduction

Atopic dermatitis (AD) is the most common inflammatory skin disease in children, especially in young children. The prevalence of childhood AD has substantially increased in many countries [1]. This increase has been too rapid to be explained by genetic changes and changes in environmental factors have been suggested as the possible explanation for the increased prevalence of AD [2].

There are many factors demonstrated to be associated with AD: personal factors (e.g., smoking habits, age, gender, nutritional status, number of siblings, lifestyle, allergy status, family history and occupation) [2] and indoor/outdoor environmental factors (e.g., house dust mite, animal dander, molds, cockroach infestation, occupational exposure, environmental tobacco smoke (ETS), air pollution, aeroallergens and climate) [3,4,5]. Many reports on indoor/outdoor environment in relation to AD were from Western populations [2,3,4,5,6] and little information on environmental risk factors associated with AD in Chinese children is available.

Recently, a large cross-sectional study has evaluated the prevalence of AD in pre-school children living in Shanghai. The study found a high prevalence of AD (8.3%) in this age group [7]. The main aim of this study was to further explore the different risk factors (e.g., home environment and personal living environment) associated with AD. Our main hypothesis is that AD is associated with residential indoor environmental factors and the urban environment in Shanghai.

## 2. Materials and Methods

### 2.1. Study Population

**Figure 1:**
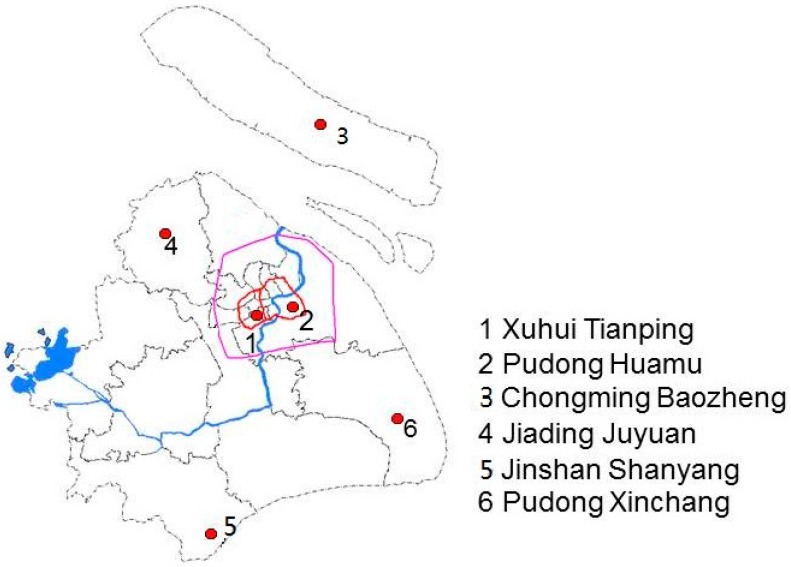
Map of the participating communities in urban and suburban/rural areas of Shanghai (the label of each community consists of the administrative district name and the community name).

The survey was conducted in six arbitrarily selected administrative districts (out of a total of 15 districts/counties) in Shanghai from April to June in 2010. In each district, one or two communities were randomly selected (Figure 1). The selected communities included two urban ones (Nos. 1–2, Figure 1) and four suburban or rural areas (Nos. 3–6). In each community, there were usually 2–3 kindergartens and a total of 20 kindergartens were recruited. The deans of all kindergartens were contacted and they all agreed to participate the survey. In each kindergarten, all children aged 3–6 years were invited and paper consents were obtained from children’s parents or guardians before the survey.

Figure 1 shows the locations of the six communities in urban and suburban/rural areas in this study. The Inner Ring surrounds the urban area of Shanghai (No. 1 and No. 2) and the Outer Ring surrounds the suburban area of Shanghai. Community No. 4 is located approximately 30 kilometers away from the center of the city; communities No. 3, No. 5 and No. 6 are approximately 60 to 100 kilometers away from the center. No. 3 is located on the island, north-east of Shanghai.

### 2.2. Questionnaire Data

A self-administered questionnaire was distributed to the children’s parents or guardians. It included questions on: (1) the children’s age, gender and early childhood environment (e.g., breastfeeding, parents-grandparents’ atopic diseases, parents’ educational level and self-reported home income level); (2) the children’s medical history including AD, history of allergic asthma and allergic rhinitis, and prevalence of food allergies; and (3) the home environment, including prenatal and current home environment.

### 2.3. Information on Parents and Grandparents

Parents’ educational level referred to parents’ highest academic level and was categorized into (1) both parents had college-level education or (2) one parent did not have college-level education. The self-reported home income level was divided into three levels: low, middle and high. One question asked whether parents or grandparents had any atopic disease, including a history of AD, allergic asthma and allergic rhinitis.

### 2.4. Early Life Environment and Allergy

One question addressed whether children were breastfed for more than 6 months or not. Questions on preterm birth, low birth weight and maternal active smoking were included. One question asked about food allergy/food intolerance in the child, with specific questions on allergy to the following foods: egg, milk, nuts, peanuts, soybean, wheat, sea food, chocolate, yoghurt, mango and pineapple. Positive answers to any of the listed food items were indicative of the presence of food allergy. In addition there was one open question on other types of food allergies. Allergy to other types of food, not specified in the questionnaire, was also counted as the presence of food allergy/intolerance (e.g., tomato and peach).

The questions on home environment addressed the prenatal and postnatal indoor environment (e.g., home renovations/decorations, maternal passive smoking, pet-keeping, *etc.*) and the current home environment (e.g., floor and wall materials, children’s passive smoking, home plants, home mold and pesticide use). The prenatal time referred to the time period 1 year before pregnancy and during pregnancy. The postnatal time referred to the period since children’s birth (from birth until the day the questionnaire was answered). For prenatal renovation/redecoration, the specific question was “Had the mother lived in a newly (*i.e.*, within 6 months after renovation) renovated/decorated home 1 year before or during pregnancy?” For prenatal exposure to new furniture, the question was “Has there been any new furniture in the home 6 months before pregnancy until now, and with a pungent odor for more than 2 months?” For the indoor painting the question was “Has there been any new painting at home since 1 year before pregnancy until now?”.

For the current home environment there were questions as follows: for indoor molds “Are there any signs of mold or mold growth on the walls at home?”; for floor material, “What types of floor materials at home?” (5 questions): (1) laminated or wood floor; (2) door-to-door carpeting; (3) tiles; (4) concrete; (5) others; for wall materials: “What types of wall materials at home?” (6 questions): (1) oil-based or water-based painting; (2) wallpaper; (3) wood; (4) tiles; (5) concrete; (6) others. Finally, one question asked about current pets in the home, including cats and dogs.

### 2.5. AD Diagnosis

The AD diagnosis were based on the U.K. Working Party’s (UKWP) criteria [8], requiring the major criterion and at least three of the five minor criteria to be fulfilled for positive diagnosis. The major criterion was presence of any itchy skin condition (*i.e.*, scratching or rubbing) in the child’s lifetime. The minor criteria included:

1. A history of skin creases in the child such as folds in the elbows, behind the knees, front of ankles or around the neck (including cheeks in children <10 years of age);
2. A history of asthma or hay fever in the child during the first four years of life (or a history of atopic disease in father or mother).
3. A history of dry skin conditions in the child the last 12 months.
4. Visible flexural dermatitis in the child (or dermatitis involving cheeks/forehead and outer areas of limbs in children under 4 years old);
5. Onset of AD in the child the first two years of life.

AD diagnosis was determined by the answers in the questionnaire. Children were identified as having AD if the answers fulfilled the above major criteria and had three or more of the minor criteria [7].

### 2.6. Statistical Analyses

The database was created by double independent data entry using the Epidata 3.1 software package (The EpiData Association, Odense, Denmark). The proportions of reports on risk factors in those with and without AD were compared by Chi-square test and differences in mean age were tested by Students *t*-test. Associations between AD and different risk factors were further evaluated by multiple logistic regression models (Enter method) with adjusted odds ratio (AOR) and a 95% confidence interval (95% CI) as the evaluation method. The regression analyses were performed in the total material and in children with and without parents’ or grandparents’ history of atopic diseases. Interaction effects between parents-grandparents’ atopic diseases (heredity susceptibility) and the environmental factors, with respect to AD were further tested by adding an interaction term in the multiple logistic regression models. Any missing values in the model variables were excluded in the final analyses. In all statistical analyses, a two-tailed test was used and the significance level was 5%. The analyses were performed by SPSS 15.0 (Chicago, IL, USA).

### 2.7. Ethic Statement

The study protocol was approved by the Ethical Committee of Huashan Hospital at Fudan University in Shanghai, China (Nr. 164). It complied with the principles outlined in the Helsinki Declaration. Informed written consent was obtained before administering the questionnaire survey.

## 3. Results

Totally 6650 children were invited to participate in the study and 6624 completed and returned the questionnaires (response rate, 99.6%). Boys accounted for 51.3%. The mean age (SD) was 5.2 ± 0.9 years old (yrs) with 11.9% at 3 yrs, 31.9% at 4 yrs, 35.0% at 5 yrs and 21.2% at 6 yrs. Data on subjects’ demographic and environmental risk factors are given in Table 1.

**Figure 2:**
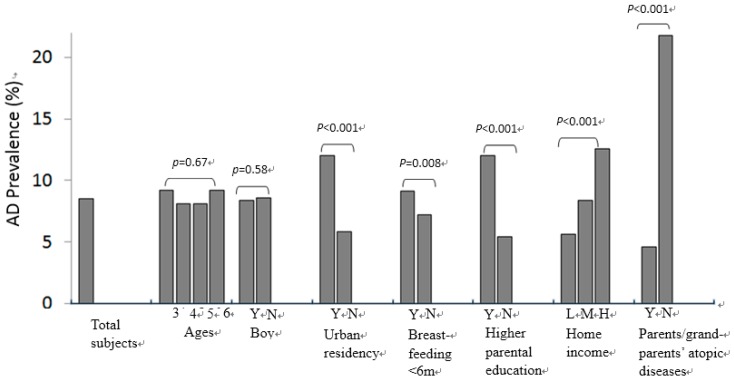
Comparisons of AD prevalence between ages, gender, residency area, breastfeeding, parental educational level, home economy and parents/grandparents’ history of atopic diseases. Y = yes; N = no. Higher parental education (Y = both parents with college-level education) *vs.* lower parental education (N = either one parent did not have college-level education); Home economy (H = higher, M = middle, L = lower).

AD was diagnosed in 8.5% of the children and 22.3% of the parents or grandparents had a history of atopic diseases (Figure 2). A higher prevalence of AD was found in children who had parents or grandparents’ atopic disease (21.8% *vs.* 4.6%, *p* < 0.001). Moreover, AD was more common in children living in urban areas (12.0% *vs.* 5.8%, *p* < 0.001), who had been breastfed for a shorter time (less than 6 months) (9.1% *vs.* 7.2%, *p* = 0.008), whose both parents had college or university education (12.0% *vs.* 5.0%, *p* < 0.001) and in children from high-income level families as compared to those from middle- or low-income level families (12.6% *vs.* 8.4% *vs.* 5.6%, *p* < 0.001) (Figure 2).

By comparing the distribution of home environmental risk factors in subjects with and without AD (Table 1), the results revealed that new home renovation/decoration was positively associated with higher prevalence of AD, both for the prenatal and postnatal period. New indoor painting and new furniture since 6 months before pregnancy, maternal passive smoking during the prenatal period, use of laminated wood or carpeted floors, home indoor molds and children’s food allergy were positively associated with AD.

By multiple logistic regression analyses (Table 2), living in urban areas currently (AOR = 1.52; 95% CI = 1.18–1.96), parents/grandparents’ history of atopic diseases (AOR 3.85; 95% CI 3.05–4.87) and children’s food allergy (AOR 3.40; 95% CI 2.63–4.40) were significantly associated with AD. Among home environmental factors, living in newly renovated/decorated homes during the prenatal period (AOR 1.41; 95% CI 1.03–1.93) and presence of indoor molds in the current dwelling (AOR 2.00; 95% CI 1.48–2.70) were significantly associated with AD.

In order to analyze the effects of heredity on childhood AD, stratified multiple logistic regression analyses were performed in two subgroups with and without parents/grandparents’ atopic diseases, respectively (Table 2). The results showed that the significant associations between AD and living in urban areas, newly renovated/decorated homes during the prenatal period and getting new furniture in the home were only found in children without a family history of atopic diseases. The significant associations between AD and food allergy as well as indoor mold in the current home were present in both groups (Table 2). Further by interaction test between parents/grandparents history of atopic diseases and significant environmental factors, new home decoration in prenatal time period showed a significant interaction effect (*p* < 0.05) (data not shown).

To explore the individual and combined effects of genetics and home environment on childhood AD, multiple logistic regression analyses were performed in four subgroups (Table 3). The reference group consisted of children with neither the specific home risk factor nor parents/grandparents’ history of atopic disease. The other three groups were either with the specific home factor only, or with parents or grandparents’ history of atopic diseases only or with both of them. The results showed that, compared with the reference group, significant positive associations were found in all other 3 subgroups. The highest AORs were found in the group with both the home risk factor and a family history of atopic disease.

## 4. Discussion

In this study, prenatal exposure to a newly renovated or redecorated home, presence of new furniture at home, currently living in a home with indoor mold were significantly associated with childhood AD in Shanghai. Food allergy, urban residency and parents/grandparents history of atopic disease were significant risk factors as well. Parents/grandparents’ history of atopic disease was an important risk factor but the environmental risk factors were more strongly associated with AD in children without parents/grandparents’ atopic diseases.

Selection bias may exist in the cross-sectional epidemiological study. We arbitrarily selected kindergartens from both urban and suburban/rural areas. The sample size was large and the response rate was high. Moreover, the participants were recruited without any prior information on children’s dermal health. Thus selection bias is fairly unlikely in this study. The diagnosis of AD was based on questionnaire data and strictly followed the diagnosis criteria of the UK working party’s criteria. These criteria have been suggested to be applicable in large-scale epidemiology studies [8] and a validation study of AD has been performed in Chinese population showing a good correlation with clinical standards with high sensitivity and specificity [9]. The same questions on AD have been used in a previous prevalence study of AD in children in Shanghai [7]. So severe information bias due to misclassification of AD is unlikely.

In Chinese cities, new home renovation/decoration and new furniture is common among young couples who are married and expecting babies. In this study, we defined the “new” home renovation/decoration in the time period “within 6 months after home renovation” in order to capture the highly elevated emissions of chemical compounds. Chemical compounds, such as formaldehyde, volatile organic compounds (VOC), benzene, toluene and xylene (BTX) and semi-volatile organic compounds (SVOC), are likely to be emitted from chipboards, polymers and newly painted surfaces [10]. A study by Xu *et al.* measured total VOC (TVOC) levels in Chinese homes within one year after renovation/decoration. The average TVOC level in 982 residential homes was 2.18 ± 12.9 mg/m^3^ [11]. This average level was much higher than the Chinese Indoor Air Quality Standard (GB/T 18883-2002) [12] of 0.6 mg/m^3^ (8 h average). Thus, we can expect that those living in newly renovated/decorated homes are likely to be exposed to elevated levels of these chemicals.

There are studies reporting an association between exposure to renovation-related pollutants, including formaldehyde [13] and VOC and atopic disorders [14,15,16]. A study by Herbarth *et al.* [15] investigated the effects of home renovation (e.g., painting, new floor covering and new furniture) at different exposure periods on the presence of eczema during childhood (mean age 6.3 ± 0.6 yrs) and later in life. Home renovations before birth (*i.e.*, prenatal) and during the child’s first year of life (early life exposure) had stronger associations with eczema compared to renovations when the children were at 2–3 years old. This is consistent with our findings of the significant associations between newly renovated homes during the prenatal period and childhood AD. A recent study found that there were significant higher concentrations of IL-4 and IL-5 in cord blood of neonates who had been exposed to renovation materials (e.g., floor covering) and new furniture [17]. VOCs are transferred from the placenta to the fetus and can affect the neonatal immune system by elevating IL-production and reducing the production of interferon [18,19]. These studies provided biochemical evidence consistent with our findings in children. On the other hand, the significant associations between home new painting and AD were not significant in the multivariate regression analyses in this study. This could be due to, at least in part, the correlation between new painting and new furniture. Since Chinese people usually have their homes painted and decorated/renovated at the same time it is difficult to separate the effect of indoor painting from the effect of other new materials used in redecoration or renovation. Further studies are needed to differentiate the effects of new painting, new furniture and home decoration.

Home renovation/decoration during the prenatal period was significantly associated with AD only in subjects without parents/grandparents’ atopic diseases. We thought the lack of association in children with parents/grandparents’ atopic diseases could be due to parents’ avoidance behavior since they are more aware and concerned on the harmful effects. However, by comparing the positive reports on living in newly renovated homes during the prenatal period in two groups with or without parents/grandparents’ atopic diseases (14.5% *vs.* 8.1%, *p* < 0.01), we found no evidence of such avoidance behavior. Further, the significant interaction effect between renovation/decoration during the prenatal period and parents/grandparents’ atopic disease indicated the potential different mechanisms of effects by renovation under different genetic characteristics (interaction term OR = 0.46; *p* < 0.05).

Presence of indoor molds in the current home was associated with childhood AD in our study and the OR was higher in children with parents/grandparents’ atopic disease. In the presence of both factors (*i.e.*, home mold and parents/grandparents’ atopic diseases), the adjusted OR for childhood AD increased to 8.38 (95% CI, 5.53–12.68) (Table 3), which was higher than the predicted ORs (*i.e.*, exposed to home mold without parents/grandparents’ atopic diseases + not exposed to home mold but with parents/grandparents’ atopic diseases; predicted OR: (1.73 – 1) + (3.64 – 1) + 1 = 4.37). This suggested an additive or synergic effect of indoor mold and the heredity susceptibility (indicated by parents/grandparents’ atopic diseases). A positive association between indoor mold exposure and childhood AD (or eczema) has been reported in other studies [19,20,21,22]. Although the exact mechanisms are unknown, mold exposure can induce a Th2-type response [23] and increase the IgE level [24,25]. Shanghai is a coastal city with a subtropical and humid climate which favor mold growth. It is suggestive that reducing indoor mold contamination in homes may reduce the risks of childhood atopic dermatitis.

In this study, food allergies were found in approximately 11% of the children and more than 30% of children with AD had food allergies. Food allergy was consistently associated with AD in the total material as well as in subgroups with and without heredity susceptibility. The close relationship between food allergy and childhood AD indicated the potentially similar immunological mechanisms which deserves further investigation [26]. Living in urban areas indicate a more westernized life style and diet, with less exposure to farms or rural areas, and could lead to more use of chemicals [27]; these factors have been demonstrated to be associated with atopic diseases in children in other studies as well [3,21].

There are several limitations in this study. Firstly, the significant associations observed in this cross-sectional study were not indicators for any cause-effect relationship. Secondly, due on the awareness of risk factors in relation to childhood AD, parents avoidance behavior might show a “protective“ effects of certain factors(“reverse causation”). Although we did not detect any significant “protective“ factors, such results need to be explained with caution.

## 5. Conclusions

Home renovation/decoration in prenatal period, new furniture and indoor molds at home are risk factors for childhood AD in Shanghai. Other risk factors include current urban residency, food allergy and parents/grandparents’ atopic disease. Preventive measures reducing the exposure to home risk factors might benefit in lowering the risk of AD in children.

## Figures and Tables

**Table 1:** Prevalence of AD and proportions of different factors in total subjects and in subjects with and without AD.

AD, Demographic and Environmental Factors	Total (*n* = 6624)	With AD (*n* = 560)	Without AD (*n* = 6064)
**AD, asthma/rhinitis and family atopic history (prevalence)**			
AD	8.5	–	–
Doctors’ diagnosed asthma or allergic rhinitis	13.3	30.0 ***	11.7
Reports on food allergy ^†^	11.6	32.3 ***	9.7
**Demographics and personal factors**			
Age (mean ± SD)	5.2 ± 0.9	5.2 ± 0.9	5.2 ± 0.9
Boy	51.3	50.7	51.4
Preterm birth	5.7	5.5	5.7
Low birth weight	3.2	3.3	3.1
**Home environmental factors**			
**Prenatal** ^#^			
Live in newly renovated/decorated home	10.2	16.2 ***	9.7
New indoor painting 6 months before pregnancy	13.0	18.3 ***	12.6
New furniture 6 months before pregnancy	9.5	17.6 ***	8.8
Pet-keeping	9.9	10.7	9.8
Maternal passive smoking	24.1	28.1 *	23.7
Maternal active smoking	0.2	0.2	0.2
**Postnatal** ^#^			
Live in newly renovated/decorated home	13.3	18.4 **	12.9
Pet-keeping	13.2	13.1	13.3
Children passive smoking	41.8	42.5	41.8
Maternal active smoking	1.1	1.3	1.1
**Current**			
Laminated wood floor/carpeting	86.0	92.1 ***	85.5
Painting, wallpaper, wood walls	98.0	99.0	97.9
Flowers or pot plants in present home	34.4	38.8 *	34.0
Daily opening of windows	97.4	97.2	97.4
Self-reported high ventilation level at home	93.0	93.6	93.0
Indoor molds	10.7	18.6 ***	10.0
House mouse or cockroach often seen	13.7	13.7	13.7
Pesticide use	18.3	12.7 ***	18.8

^#^ Prenatal time refers to the 1 year before pregnancy and during pregnancy; postnatal time refers to the time period from children’s birth until now; † Self-reported food allergies to eggs, milk, nuts, peanuts, soybean, wheat, sea food, chocolate, yoghurt, mango, pineapple, tomato, peach *etc*. * *p* < 0.05, ** *p* < 0.01, *** *p* < 0.005.

**Table 2:** Association (AORs and 95% CI) between childhood AD and demographic and home environmental factors in total subjects and subgroups with and without parents/grandparents’ atopic diseases ^†^.

Demographic and Environmental Factors	Total (*n* = 5088)	Without Parents/Grandparents’ Atopic Diseases (*n* = 3942)	With Parents/Grandparents’ Atopic Diseases (*n* = 1146)
**Demographic and personal factors**			
Parents/grandparents’ atopic diseases	3.85 (3.05–4.87) ***	–	–
Food allergy	3.40 (2.63–4.40) ***	4.44 (3.05–6.47) ***	2.73 (1.93–3.87) ***
Age	1.05 (0.93–1.20)	1.02 (0.85–1.22)	1.09 (0.91–1.30)
Boy	0.95 (0.76–1.19)	1.03 (0.75–1.43)	0.88 (0.64–1.20)
Live in urban areas currently	1.52 (1.18–1.96) **	1.99 (1.39–2.85) ***	1.11 (0.78–1.59)
Breastfeeding less than 6 months	1.02 (0.81–1.29)	1.15 (0.82–1.61)	0.90 (0.65–1.25)
Self-reported higher income level	1.28 (0.95–1.73)	1.40 (0.91–2.18)	1.16 (0.77–1.76)
Higher parental educational level	1.22 (0.93–1.61)	1.20 (0.82–1.75)	1.25 (0.84–1.87)
**Home environmental factors**			
**Prenatal**			
Live in newly renovated/decorated home	1.41 (1.03–1.93) *	2.10 (1.35–3.26) **	0.98 (0.63–1.52)
Home new painting since 6 m before pregnancy	1.05 (0.76–1.44)	0.77 (0.46–1.30)	1.35 (0.90–2.05)
Home new furniture since 6 m before pregnancy	1.37 (0.98–1.93)	1.91 (1.17–3.14) *	1.07 (0.68–1.67)
Maternal passive smoking	0.97 (0.73–1.29)	1.01 (0.66–1.52)	0.94 (0.64–1.39)
**Postnatal**			
Live in newly renovated/decorated home	1.09 (0.80–1.50)	1.09 (0.67–1.77)	1.10 (0.72–1.67)
Children passive smoking	1.00 (0.77–1.31)	1.15 (0.79–1.67)	0.86 (0.59–1.25)
**Current**			
Laminated, wood or carpeting floor	1.29 (0.80–2.07)	1.24 (0.67–2.28)	1.26 (0.57–2.76)
Home flowers or plants	1.14 (0.90–1.44)	1.05 (0.75–1.47)	1.19 (0.86–1.64)
Indoor molds	2.00 (1.48–2.70) ***	1.64 (1.04–2.60) *	2.32 (1.54–3.49) ***
Pesticide often used	0.72 (0.51–1.02)	0.64 (0.38–1.06)	0.78 (0.48–1.26)

* *p* < 0.05; ** *p* < 0.01; *** *p* < 0.001; ^†^ Multivariate logistic regression analyses were performed including all variables listed above which showed positive effects on AD in the Chi-x^2^ test. The number of subjects might not be equal to the total subjects due to missing values.

**Table 3:** The individual and combined effects (AOR, 95%CI) of home risk factors and parents/grandparents’ atopic diseases on childhood AD ^†^.

Subgroups	(Pre) New Decoration ^‡^	Wall Molds	New Furniture
No environmental exposure and no parents/grandparents’ atopic diseases (ref)	1	1	1
Environmental exposure but no parents/grandparents’ atopic diseases	2.11 (1.38–3.21)	1.73 (1.10–2.70)	1.90 (1.19–3.01)
No environmental exposure but parents/grandparents’ atopic diseases	4.40 (3.40–5.68)	3.64 (2.84–3.75)	4.22 (3.28–5.44)
Both environmental exposure and parents/grandparents’ atopic diseases	4.26 (2.72–6.68)	8.38 (5.53–12.68)	4.50 (2.86–7.06)

^†^ AOR (95% CI) were estimated by logistic regression adjusting for age, gender, breastfeeding, parental educational level, living area (urban/sub-rural), family economy level, newly decorated homes during prenatal period (for wall mold variable and new furniture), postnatal living in newly decorated homes, new home painting, new home furniture (for new decoration during prenatal period and wall molds), maternal passive smoking, children passive smoking, floor material, home plants, mold on walls (home new decoration during prenatal period and new furniture), pesticide use and food allergy; ^‡^ “(pre) new decoration” refers to living in newly decorated/renovated homes 1 year before pregnancy and during pregnancy.
